# Prevention of hypoparathyroidism: A step-by-step near-infrared autofluorescence parathyroid identification method

**DOI:** 10.3389/fendo.2023.1086367

**Published:** 2023-01-30

**Authors:** Junwei Huang, Yurong He, Yuan Wang, Xiao Chen, Yang Zhang, Xiaohong Chen, Zhigang Huang, Jugao Fang, Qi Zhong

**Affiliations:** Key Laboratory of Otolaryngology Head and Neck Surgery (Capital Medical University), Department of Otorhinolaryngology Head and Neck Surgery, Beijing Tongren Hospital, Capital Medical University, Ministry of Education, Beijing, China

**Keywords:** thyroid cancer, thyroidectomy, hypoparathyroidism, near-infrared autofluorescence, parathyroid location

## Abstract

**Background:**

Hypoparathyroidism is an important factor that seriously affects the quality of life of patients after thyroidectomy. This study aimed to optimize the surgical procedure for parathyroid identification using near-infrared autofluorescence (NIRAF) during thyroidectomy.

**Methods:**

This was a prospective controlled study that included 100 patients with primary papillary thyroid carcinoma diagnosed in Beijing Tongren Hospital between June 2021 and April 2022 who were awaiting total thyroidectomy and bilateral neck dissection. The patients were randomly divided into an experimental group in whom step-by-step NIRAF imaging was used to identify parathyroid glands, and a control group in whom NIRAF was not used.

**Results:**

The number of parathyroid glands identified in the NIRAF group was higher than that in the control group (195 vs. 161, p=0.000, Z=-5.186). The proportion of patients with parathyroid glands inadvertently removed in the NIRAF group was lower than that in the control group (2.0% vs. 18.0%, respectively; p=0.008, χ^2 =^ 7.111). In the NIRAF group, we found that more than 95% of the superior parathyroid glands and more than 85% of the inferior parathyroid glands were identified before the dangerous phase, which was much higher than that in the control group. The incidences of temporary hypoparathyroidism, hypocalcemia, and symptomatic hypocalcemia were higher in the control group than those in the NIRAF group. On the first postoperative day, the average parathyroid hormone (PTH) level in the NIRAF group decreased to 38.1% of the preoperative level and that in the control group decreased to 20.0% of the preoperative level (p=0.000, Z=-3.547). On the third postoperative day, the PTH level in 74% of the patients in the NIRAF group recovered to normal levels, whereas it recovered in only 38% of the patients in the control group (p=0.000, χ^2 =^ 13.149). The PTH levels in all patients in the NIRAF group had recovered within 30 days after surgery, whereas one patient in the control group failed to return to the normal level 6 months after surgery and was diagnosed with permanent parathyroidism.

**Conclusions:**

The step-by-step NIRAF parathyroid identification method can effectively locate the parathyroid gland and protect its function.

## 1 Introduction

Over the past 50 years, the incidence of thyroid cancer has been rising globally, especially in countries with middle- and high-income populations and it will soon become the fourth most common cancer worldwide (1). In China, thyroid cancer has become one of the fastest-growing malignant conditions, and its age-standardized incidence increased to 9.61/100,000 in 2015 (2, 3). However, since more than 90% of thyroid cancers are of the differentiated type, the vast majority (>85%) of patients survive for a long time through surgery, postoperative thyroid stimulating hormone (TSH) suppression therapy, and radioactive iodine therapy (4). Hence, more attention has been paid to postoperative complications because of their impact on quality of life. It has been reported that the incidence of vocal cord paralysis caused by recurrent laryngeal nerve injury after thyroidectomy is 0.2% to 3.9%, and the incidence of permanent hypoparathyroidism is 0.1% to 8.8% (5). After total thyroidectomy, the incidence of permanent hypoparathyroidism can be as high as 12% (6). Intraoperative neuromonitoring is now the standard surgical procedure for protecting the laryngeal nerve from recurrent injuries and can significantly reduce these (7). Nevertheless, there is no ideal solution for protection of the parathyroid gland, and permanent hypoparathyroidism is an important condition that seriously affects the postoperative quality of life of patients (8).

In recent years, near-infrared autofluorescence (NIRAF) imaging has become a real-time intraoperative identification technique for the parathyroid glands. This technology was approved by the US Food and Drug Administration in 2018 (9). Its sensitivity, specificity, positive predictive value, and negative predictive value were reported to be 98.5%, 97.2%, 95.1%, and 99.1%, respectively (10). Clinical research reports and meta-analyses have confirmed its value in thyroid surgery and can increase the recognition rate of parathyroid glands and reduce the incidence of postoperative hypoparathyroidism and hypocalcemia (11, 12).

It is impossible to perform NIRAF imaging continuously during surgery because the operating lamp must be removed during exploration. We found that parathyroid glands in different positions could often be identified first at a certain stage of surgery, which was better than visual recognition in most cases. Simultaneously, good imaging depends on the camera angle. Owing to the weak penetrability of fluorescence, its usefulness is also affected when the parathyroid glands are covered by other tissues (more than 5 mm) (13).

From our research data, we developed a more effective detection process to increase the recognition rate for parathyroid glands, minimize the frequency of imaging, and improve the efficiency of intraoperative detection.

## 2 Materials and methods

### 2.1 Patients

This was a prospective controlled study (registration no. ChiCTR2100046912), and was approved by the Ethics Committee of Beijing Tongren Hospital, Capital Medical University. Patients with primary papillary thyroid carcinoma diagnosed at Beijing Tongren Hospital between June 2021 and April 2022 who were awaiting surgery were included. The study followed the Helsinki Declaration, revised in 2013, and all the patients provided signed informed consent.

The inclusion criteria were: primary papillary thyroid carcinoma diagnosed by pathology. All patients had T1-3N0-1 lesions, and imaging did not indicate distant metastasis. All patients underwent total thyroidectomy and bilateral central lymph node dissection +/- lateral neck lymph node dissection by the same treatment team. The surgical indications in this study include multiple cancer foci, bilateral cervical lymph node metastases, lateral cervical metastasis, isthmus carcinoma, T2 or T3 lesions.

The exclusion criteria were: patients under 18 years of age. Patients with other pathological types of thyroid cancer or other head and neck tumors. T4 lesion. Massive thyroid tumors which may directly invade the parathyroid gland. Preoperative imaging suggesting that the tumor exceeded the capsule and involved the surrounding structures (patients in whom the extracapsular focal invasion identified during surgery would not affect the planned surgical procedure were still included in the study). Patients with a history of thyroid surgery or radiofrequency ablation. Patients with parathyroid disease or abnormal blood calcium levels before surgery. Patients choosing non-open surgery or other intraoperative imaging methods for parathyroid glands.

All patients were divided into two groups based on random number generation before surgery. In the experimental group, NIRAF imaging was used to assist in identification of the location of the parathyroid glands at different stages of surgery. In the control group, the parathyroid glands were observed and located with the naked eye.

### 2.2 Procedure

**Figure 1:**
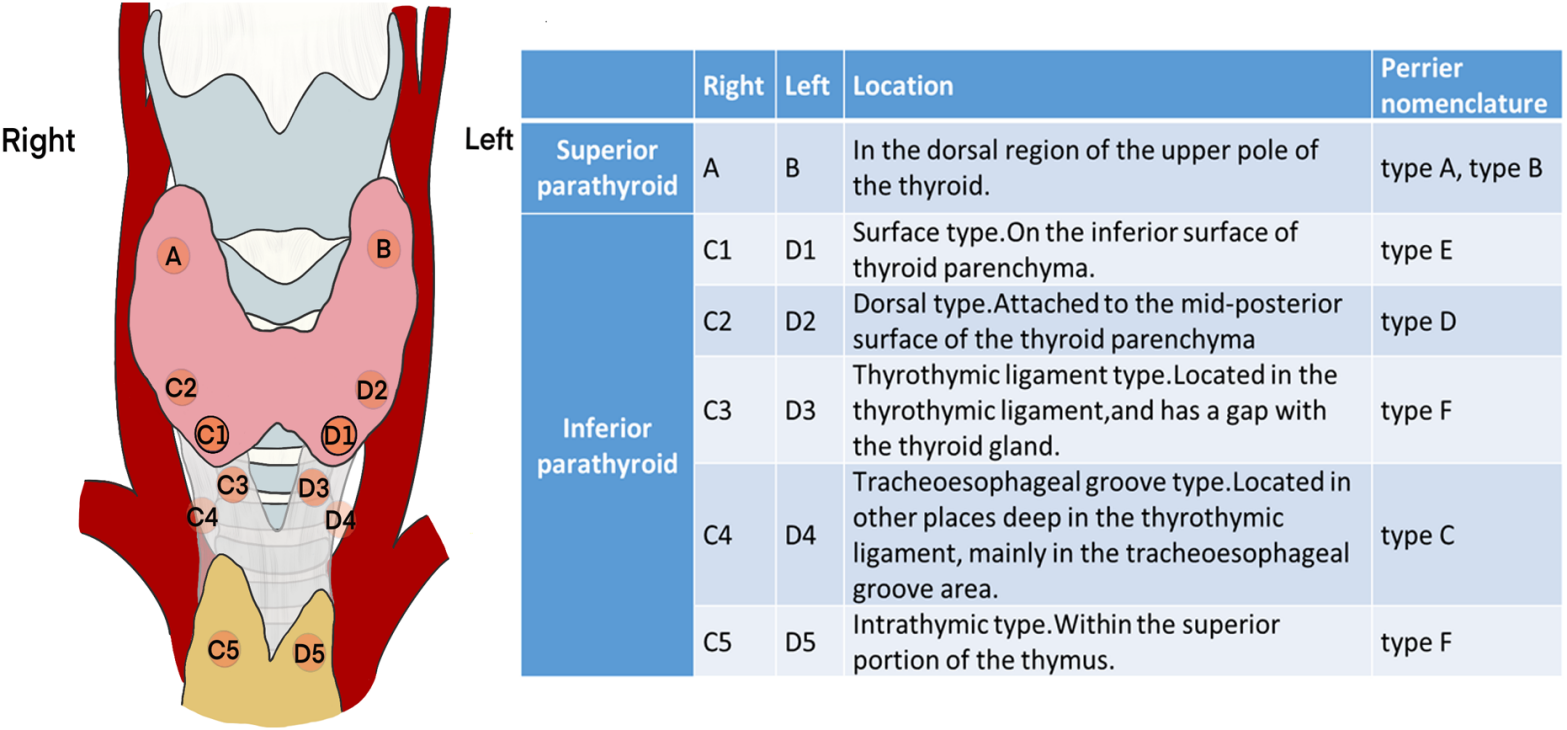
Classification and schematic diagram of the parathyroid glands according to the surgical exploration process.

Referring to the “Perrier” nomenclature (14), parathyroid glands can be roughly divided into several types based on the location and steps in the surgical procedure (**Figure 1**). Because the position of the superior parathyroid gland is relatively fixed and the deviation is small, it is no longer subdivided.

The operation can be performed from cephalic to caudal or from caudal to cephalic, in accordance with the surgeon’s preference. Because the autofluorescence signal of parathyroid glands is best when imaging in vertical direction, the imaging head of the camera must be rotated slightly during localization. To reduce the imaging frequency, the procedure can be divided into several phases (P1-P6). The following is an example from the right side.

**Figure 2:**
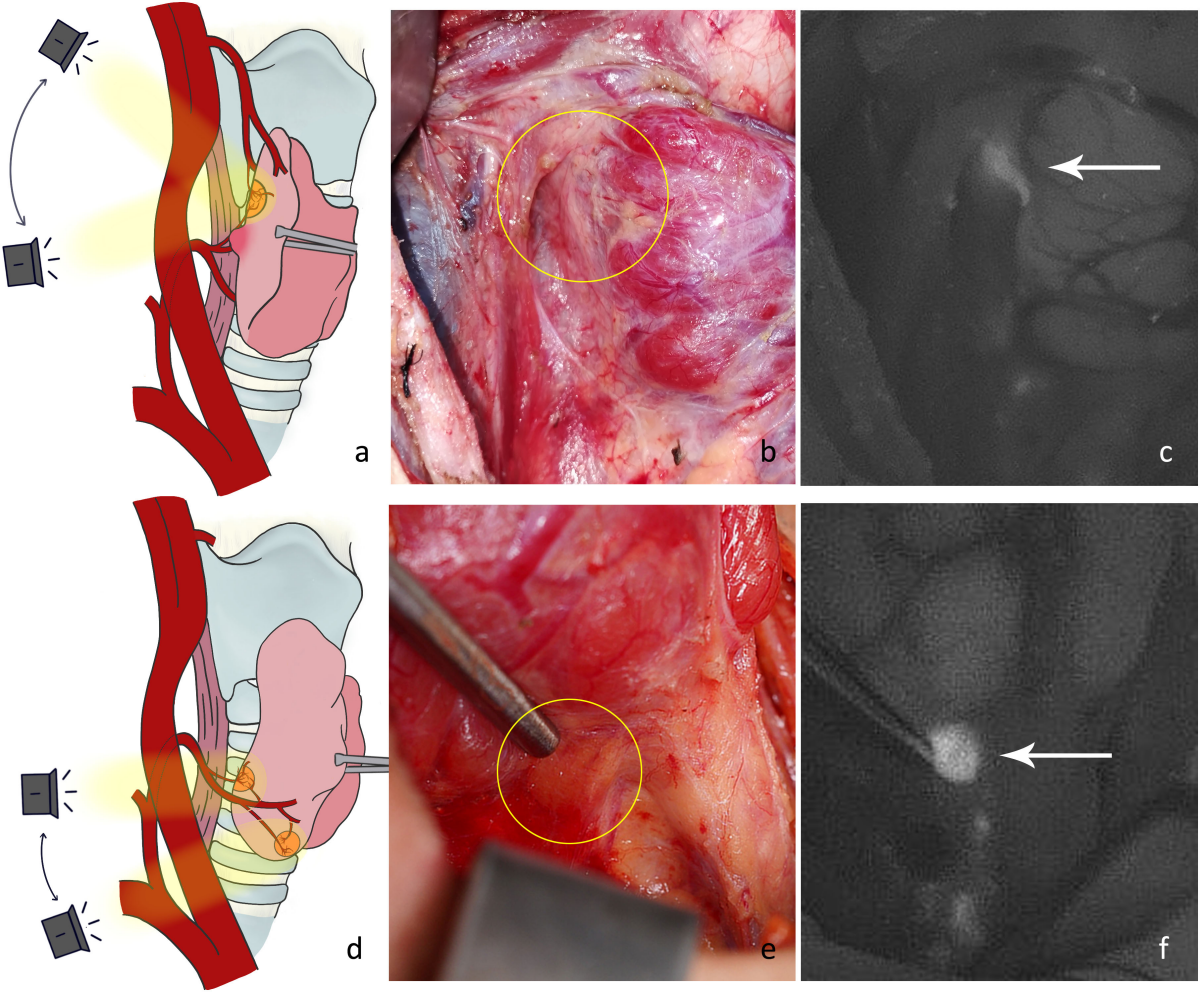
**(A)** Schematic diagram of the imaging angle for the right superior parathyroid gland. **(B)** Location of a type A gland in normal white light (yellow circle). **(C)** NIRAF showing a cordlike type A gland (white arrow). **(D)** Schematic diagram of the imaging angle for the right inferior parathyroid gland. **(E)** Location of the type C1 gland under normal white light (yellow circle). **(F)** NIRAF shows a type C1 gland (white arrow).

P1: Lateral thyroid dissection. The middle vein can be cut close to the thyroid gland to dissect the fascial tissue on the lateral side of the thyroid. Then the NIRAF camera was moved from the lateral to the medial side to capture the fluorescence signal from the parathyroid glands (**Figures 2A, D**). We mainly looked for type A superior parathyroid glands (**Figures 2B, C**), and surface type C1 (**Figures 2E, F**) or dorsal type C2 inferior parathyroid glands. Occasionally, fluorescence of the thyrothymic ligament type C3 gland could be seen through the thyrothymic ligament, yet the outline of the gland was often not clear. This could only be confirmed when the thyrothymic ligament was thin. During this phase, the blood supply to the parathyroid gland was intact.

**Figure 3:**
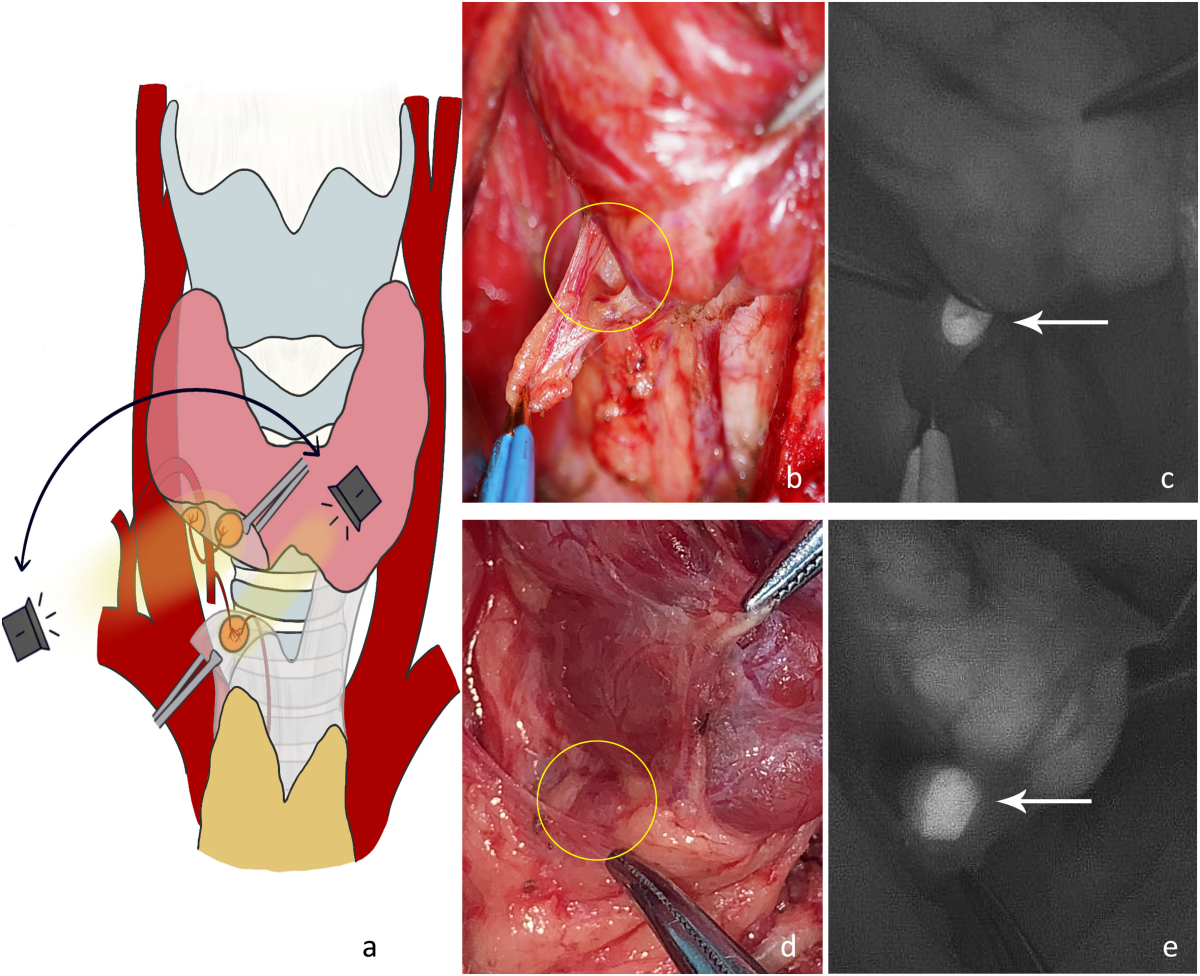
**(A)** The dorsal side of the thyroid gland and thyrothymic ligament were detected to locate the right inferior parathyroid gland. **(B)** Type C3 gland on the dorsal side of the thyrothymic ligament under normal white light (yellow circle). **(C)** The NIRAF shows a type C3 gland (white arrow). **(D)** Intrathymic type C5 gland with high position under normal white light (yellow circle). **(E)** The NIRAF shows a type C5 gland (white arrow).

P2: Dissection of the thyrothymic ligament. After separating the junction of the thyrothymic ligament and thyroid gland, the thyroid gland was pulled upward and inward. The dorsal surfaces of the thyroid and thyrothymic ligament were detected (**Figure 3A**). A type C2 gland (there was usually blood supply from the direction of the thyroid, inferior thyroid artery, or thymus) or type C3 gland (**Figures 3B, C**) (most of the blood supply was from the thymus, and some may have been combined with the inferior artery blood supply. Generally, there was no blood supply from the direction of the thyroid parenchyma.) would present substantial autofluorescence and be located. Occasionally, an intrathymic type C5 gland was located (**Figures 3D, E**). Blood supply to the inferior parathyroid gland at this stage was generally good.

**Figure 4:**
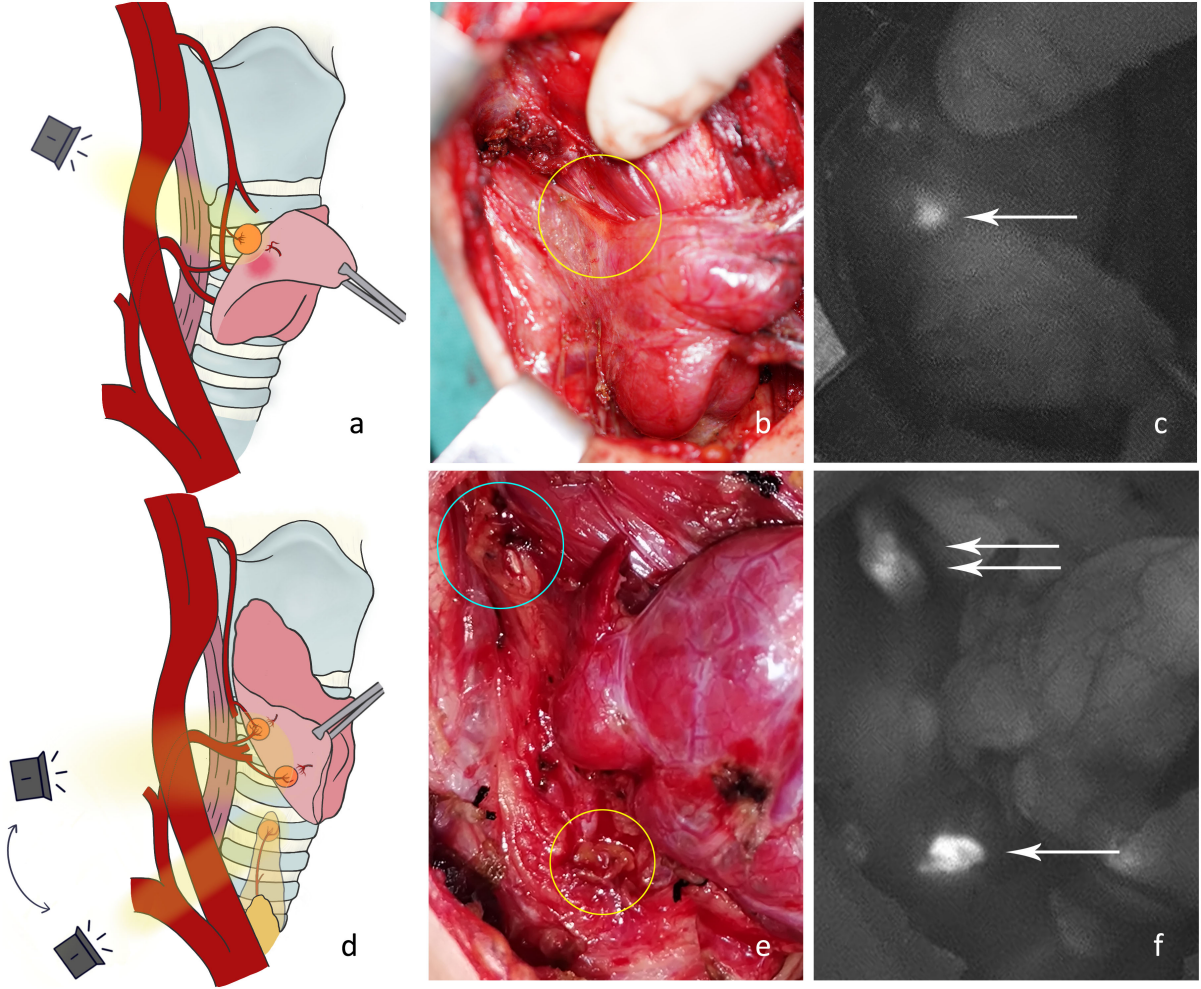
**(A)** The anterior branch of the superior thyroid artery was disconnected and the right superior parathyroid gland was detected. **(B)** The superior parathyroid gland on the dorsal side of the thyroid was difficult to see under normal white light (yellow circle). **(C)** The NIRAF shows a type A gland (white arrow). **(D)** Part of the inferior vessels was disconnected and the right inferior parathyroid gland was imaged. **(E)** The type A gland (blue circle) and type C4 gland (yellow circle) are shown under normal white light. **(F)** The NIRAF shows a type A gland (white double arrow) and a type C4 gland (white arrow).

P3: Ligation of tertiary vessels on the thyroid surface. 1) The anterior branch of the superior thyroid artery was disconnected and the superior pole of the thyroid was lifted. The superior parathyroid gland was searched from the direction of the superior blood vessel to the point where the recurrent laryngeal nerve entered the larynx (**Figures 4A–C**). At this time, the blood supply to the superior parathyroid gland from the thyroid was disconnected, and the blood supply from the posterior branch of the superior artery or from the inferior artery may still be preserved. Attention should be paid to protecting the vascular network and adipose capsule around the parathyroid gland. 2) Parts of the inferior vessels were severed close to the thyroid on the medial side of the possible inferior parathyroid gland. The inferior pole of the thyroid gland was further pulled upward to expose the deep structure. The dorsal side of the thyroid was detected from the lateral side to the medial side (**Figure 4D**). A deeper type C2 or tracheoesophageal groove-type C4 gland was detected (**Figures 4E, F**). At this time, the blood supply to the inferior parathyroid gland from the thyroid was disconnected and the blood supply from the inferior artery was intact. Therefore, the vascular network around the parathyroid glands should be protected. Generally, the blood supply in the direction of the thymus is sufficient for a type C3 gland. If the blood supply from the inferior artery can be preserved, the blood circulation can be improved.

**Figure 5:**
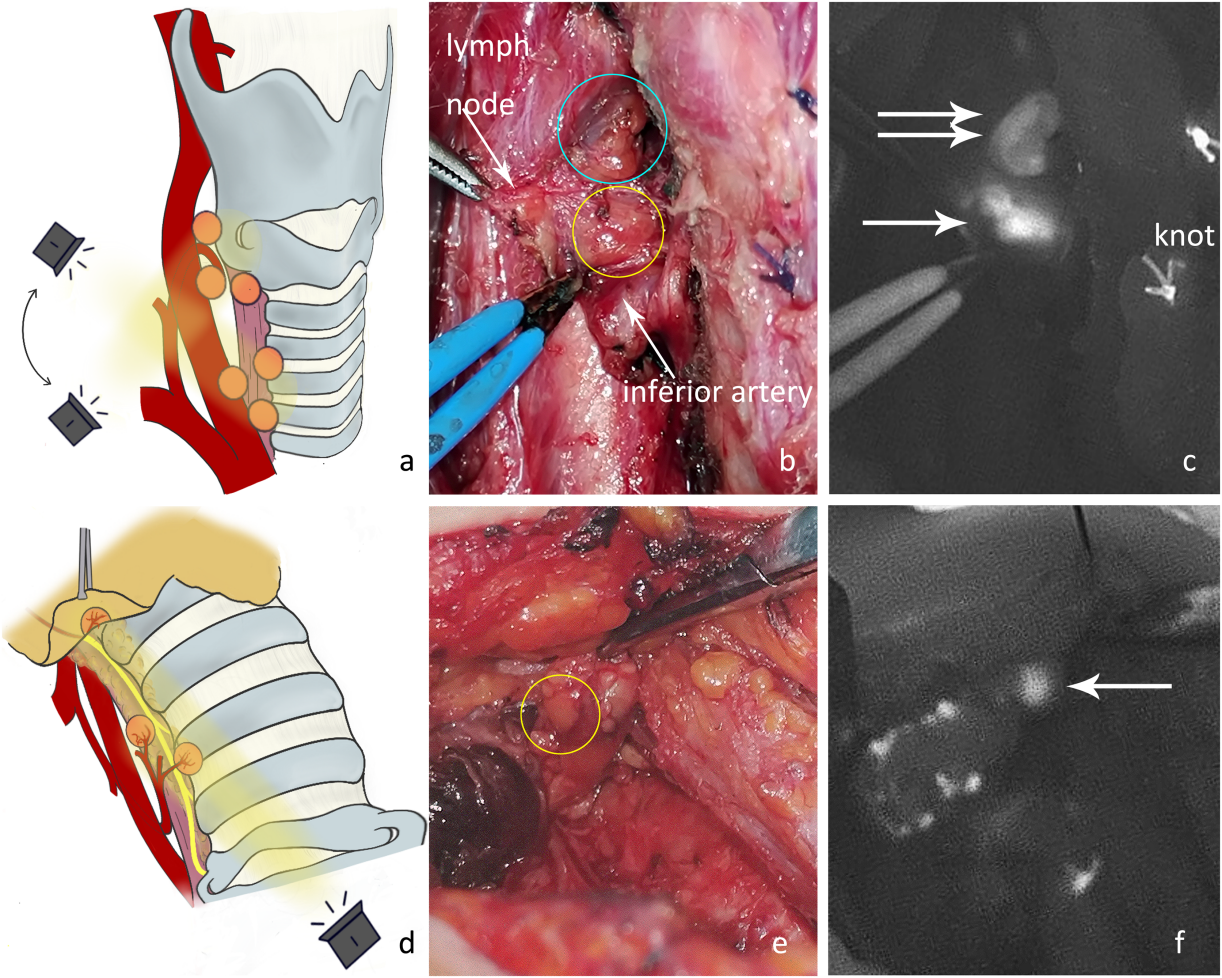
**(A)** Exploration of the deep surgical field of the thyroid bed after thyroidectomy. **(B)** Under normal white light, there were two nodules between the superior parathyroid gland (blue circle) and the inferior artery in the tracheoesophageal groove. The nodule in the yellow circle was later confirmed by NIRAF to be the inferior parathyroid gland, and its blood supply originated from the inferior artery. **(C)** The NIRAF shows that the lymph node did not develop, and the type C4 gland (white arrow) was below the superior parathyroid gland (white double arrow). **(D)** The tissue at level VI was detected after dissociating the recurrent laryngeal nerve. **(E)** The type C5 gland (yellow circle) is shown under normal white light. **(F)** The NIRAF shows a type C5 gland (white arrow).

P4: Thyroid gland removal. The surrounding tissue was then stretched, and the tracheoesophageal groove was exposed. The camera should be positioned directly above and with a search for parathyroid glands that were hidden deep in the tracheoesophageal groove or covered by other tissues (**Figure 5A**). The blood supply to the newfound superior parathyroid A or tracheoesophageal groove type C4 (**Figures 5B, C**) may have been damaged.

P5: Central neck dissection. The recurrent laryngeal nerve was dissected, and the tissues at level VI and thymus were exposed. The thymic tongue was pulled upwards with imaging from the cephalic to caudal side toward the proximal end of the recurrent laryngeal nerve (**Figure 5D**). An intrathymic type C5 (**Figures 5E, F**) or tracheoesophageal groove type C4 may be detected covered by connective tissue in level VI. Here, the blood supply to C5 in the thymus was adequate, yet the blood supply to newfound C4 in level VI may have been damaged.

**Figure 6:**
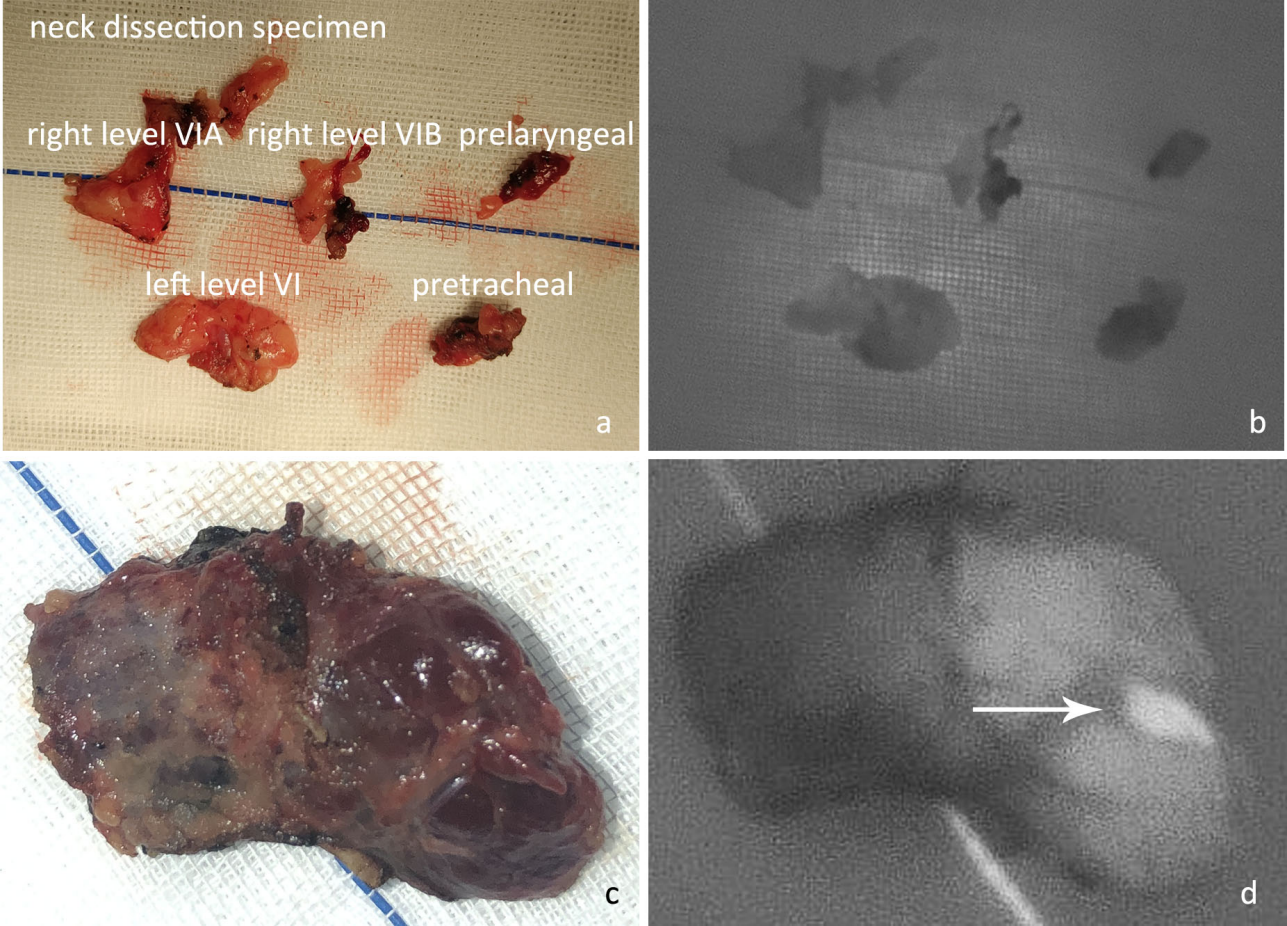
**(A)** After en bloc resection, the neck dissection specimen was examined in different regions. **(B)** The NIRAF revealed that no parathyroid gland had been accidentally resected. **(C)** Examination of excised thyroid specimens. **(D)** The isolated left superior parathyroid gland (white arrow) was found on examination of the thyroid specimen. (These two images were from a case not included in this controlled study.).

P6: Examination of isolated specimens. After the specimen was obtained, it was sent to the pathology department for frozen section or paraffin section examination. Before that, all tissues that had been removed were placed on gauze to carefully check whether there was any parathyroid gland that had been inadvertently removed (**Figure 6**).

The parathyroid gland identified in the paraffin pathology was recorded as P7. PX was recorded if no parathyroid gland was located during surgery or found in a postoperative specimen.

The NIRAF and control groups both underwent the same surgical procedure. The main difference was that, in the NIRAF group, the position of the parathyroid glands was preferentially identified, and then the surrounding blood vessels and connective tissue were retrogradely dissected towards the superior thyroid artery or inferior artery. In the control group, the parathyroid glands were identified by the naked eye during dissection from the surrounding tissue with increased risk of damaging their blood supply vessels.

### 2.3 Parathyroid development and judgement

In the NIRAF group, a handheld portable camera (Jinan Microsmart Intelligence Technology Co., Ltd. China) was used. The wavelength of the output light from the near-infrared transmitter was 785 nm. The optical power was 1 mW/cm (2), and the focal length of the collector lens was 8 mm. The imaging head of the camera was positioned approximately 10-15 cm above the surgical. The 820 nm excitation light in the surgical field was collected and imaged in black-and-white mode. The parathyroid gland may show a white oval signal that is significantly higher than that of the thyroid or the surrounding tissue in the thyroid bed. The stage and location of parathyroid exploration in each patient were recorded.

The parathyroid glands identified during surgery were jointly judged by two senior surgeons (with more than 5 years of thyroid surgical experience). If both surgeons believed that the indicated tissue was parathyroid, they would not submit it for pathological examination and it would be recorded as correct. If any doctor suspected that the indicated tissue was not parathyroid or other tissues might be parathyroid, small pieces were cut from all suspicious tissues and sent to pathology for diagnosis and exclusion of metastatic lymph nodes or fat. The parathyroid glands identified in the excised specimens or with a completely severed blood supply were autografted. All excised tissues underwent postoperative pathological examination to confirm whether there were inadvertently excised parathyroid glands.

### 2.4 Serological examination

All patients were examined for serum calcium level (normal reference range 2.20-2.65 mmol/L) and parathyroid hormone (PTH) level (normal reference range 12-88 pg/mL) before surgery. These indexes were re-checked on the first postoperative day. The occurrence of symptomatic hypocalcemia (fingertip or lip numbness, positive Chvostek sign, or tetany) was also recorded. If the symptoms were obvious or the PTH level was below the lower limit of normal, the minimum dose of oral and intravenous calcium supplementation was used in patients until they had no symptoms of hypocalcemia. If any patient had temporary hypoparathyroidism, their PTH levels were re-checked on the 3rd and 7th postoperative days and during monthly outpatient follow-up until they returned to the normal range.

### 2.5 Statistical analysis

SPSS statistical software (version 18.0; IBM Corp., Armonk, NY, USA) was used for data analysis. The chi-squared test was used to analyze the difference in count data between groups. An independent t-test was used to compare the measurement data if they had a normal distribution. The non-parametric Mann–Whitney U-test was used when the measurement data did not conform to a normal distribution using the Shapiro Wilk test. The calibration level was set at α=0.05.

## 3 Results

In total, 50 patients were included in each group (**Table 1**). In the NIRAF group, there were 195 parathyroid glands with an average of 3.9 parathyroid glands per case, whereas 161 parathyroid glands were identified in the control group, with an average of 3.2 parathyroid glands per case (p<0.001, Z=-5.186). Three patients in the NIRAF group underwent transplantation with one parathyroid gland because of a lack of blood supply, whereas 17 patients in the control group underwent transplantation with one or two parathyroid glands (p<0.001, χ^2 =^ 12.250). In postoperative paraffin pathological examination, one case in the NIRAF group was found to have an inadvertently removed parathyroid gland, whereas nine cases in the control group were found to have inadvertently removed parathyroid glands (one patient had two parathyroid glands resected) (p=0.008, χ^2 =^ 7.111) (**Table 2**).

**Table 1:** Demographics for the 100 patients with papillary thyroid carcinoma in this study.

		NIRAF group	control
sex(n)	male	13	13
	female	37	37
average age (yrs old)		47.88	43.03
stage(n)	T1	41	40
	T2	8	9
	T3	1	1
	N0	17	15
	N1	33	35

**Table 2:** Statistics for the parathyroid gland parameters in the two groups.

		NIRAF group	control
Parathyroid detected during surgery(n)	Total	195	161
	Superior parathyroid	100(51.3%)	85(52.8%)
	type A	51	41
	type B	49	44
	in paraffin section	0	4
	Inferior parathyroid	95(48.7%)	76(47.2%)
	type C1	3	1
	type C2	18	16
	type C3	15	13
	type C4	8	9
	type C5	4	2
	type D1	4	1
	type D2	16	9
	type D3	12	14
	type D4	11	8
	type D5	4	3
	in paraffin section	1	6
Inadvertently removed/Total(%)		0.5%(1/196)	5.8%(10/171)
Proportion of patients with parathyroid inadvertently removed(%)		2.0%(1/50)	18.0%(9/50)
Parathyroid transplanted during surgery (n)	Total	3	20
	Superior parathyroid	1	5
	Inferior parathyroid	2	15
	Transplanted/Total(%)	1.5%(3/196)	11.7%(20/171)
Proportion of patients with parathyroid transplantation(%)		6.0%(3/50)	34.0%(17/50)

**Figure 7:**
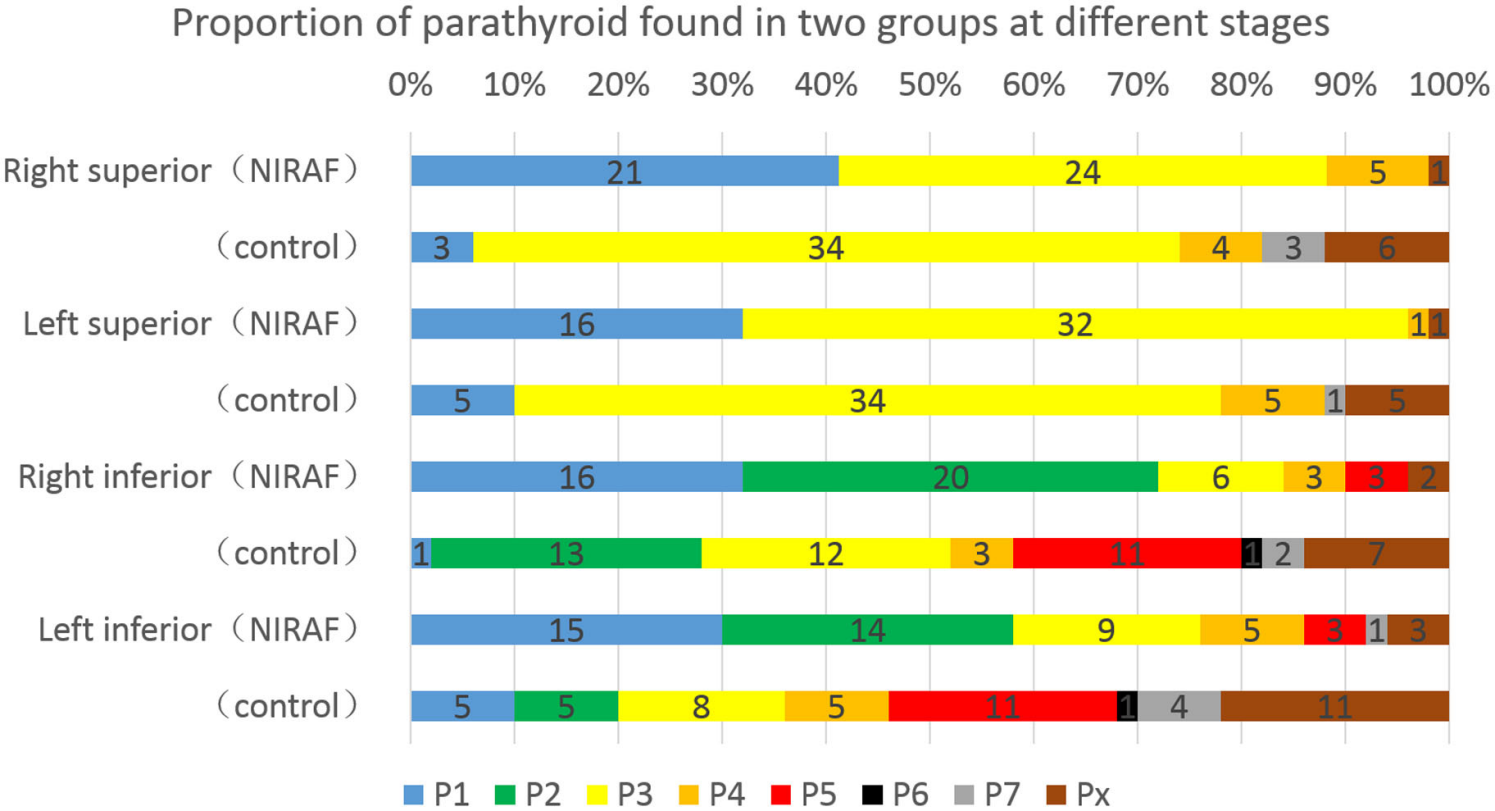
Different stages of parathyroid gland discovery in the NIRAF group and control group. Blue and green bars were basically safe phases, and the blood supply vessels in the parathyroid glands remained intact. Yellow and brownish yellow bars were the last stages of gland disconnection, and the blood supply vessels might be at risk. The red bars indicate that the blood supply to the parathyroid gland was at a critical stage during lymph node dissection. Black and gray bars indicate that the parathyroid gland had been isolated or identified in paraffin pathology. The brown bars indicate that the parathyroid gland was not located.

In the NIRAF group, 41.2% (right) and 32.0% (left) of the superior parathyroid glands were present in a safe phase (P1+P2), whereas in the control group, only 6.0% (right) and 10.0% (left) of the glands were in this phase (p<0.001, χ^2 =^ 17.245 and p=0.007, χ^2 =^ 7.294, respectively). During exploration for inferior parathyroid glands, 72.0% (right) and 58.0% (left) of the glands in the NIRAF group were in a safe phase, whereas only 28.0% (right) and 20.0% (left) of the glands in the control group were in this phase (p<0.001, χ^2 =^ 19.360 and p=0.002, χ^2 =^ 9.567, respectively). In the NIRAF group, 90.0% (right) and 86.0% (left) of the inferior parathyroid glands were detected before the dangerous phase (P5), indicating that most parathyroid glands could be retained *in situ* and had a good blood supply. In the control group, the proportion decreased significantly to 58.0% (right) and 46.0% (left) (p<0.001, χ^2 =^ 13.306 and p<0.001, χ^2 =^ 17.825, respectively) (**Figure 7**).

There was no significant difference in the mean PTH and serum calcium levels between the two groups before surgery (p=0.952, t=0.061 and p=0.676, t=-0.419, respectively). On the first postoperative day, the mean value of PTH in the NIRAF group was 21.70 pg/mL, whereas that in the control group was 11.44 pg/mL (p<0.001, Z=-3.489). The average PTH level in the NIRAF group decreased to 38.1% of the preoperative level and that in the control group decreased to 20.0% of the preoperative level (p<0.001, Z=-3.547). On the first postoperative day, the mean value of blood calcium in the NIRAF group was 2.22 mmol/L, whereas that in the control group was 2.15 mmol/L (p=0.017, Z=-2.397). The incidences of temporary hypoparathyroidism (p=0.005, χ^2 =^ 7.853), hypocalcemia (p=0.016, χ^2 =^ 5.769), and symptomatic hypocalcemia (p<0.001, χ^2 =^ 12.190) were higher in the control group than in the NIRAF group (**Table 3**).

**Table 3:** Levels of parathyroid hormone and blood calcium before and after surgery.

		NIRAF group	control
mean level of PTH	Preoperative (pg/ml)	56.91	56.70
	First postoperative day (pg/ml)	21.70	11.44
	Postoperative/Preoperative(%)	38.1%	20.0%
Incidence of temporary parathyroidism (%)		34.0%(17/50)	62.0%(31/50)
mean level of serum calcium	Preoperative (mmol/L)	2.39	2.40
	First postoperative day (mmol/L)	2.22	2.15
Incidence of hypocalcemia (%)		36.0%(18/50)	60.0%(30/50)
Incidence of symptomatic hypocalcemia(%)		14.0%(7/50)	46.0%(23/50)

**Figure 8:**
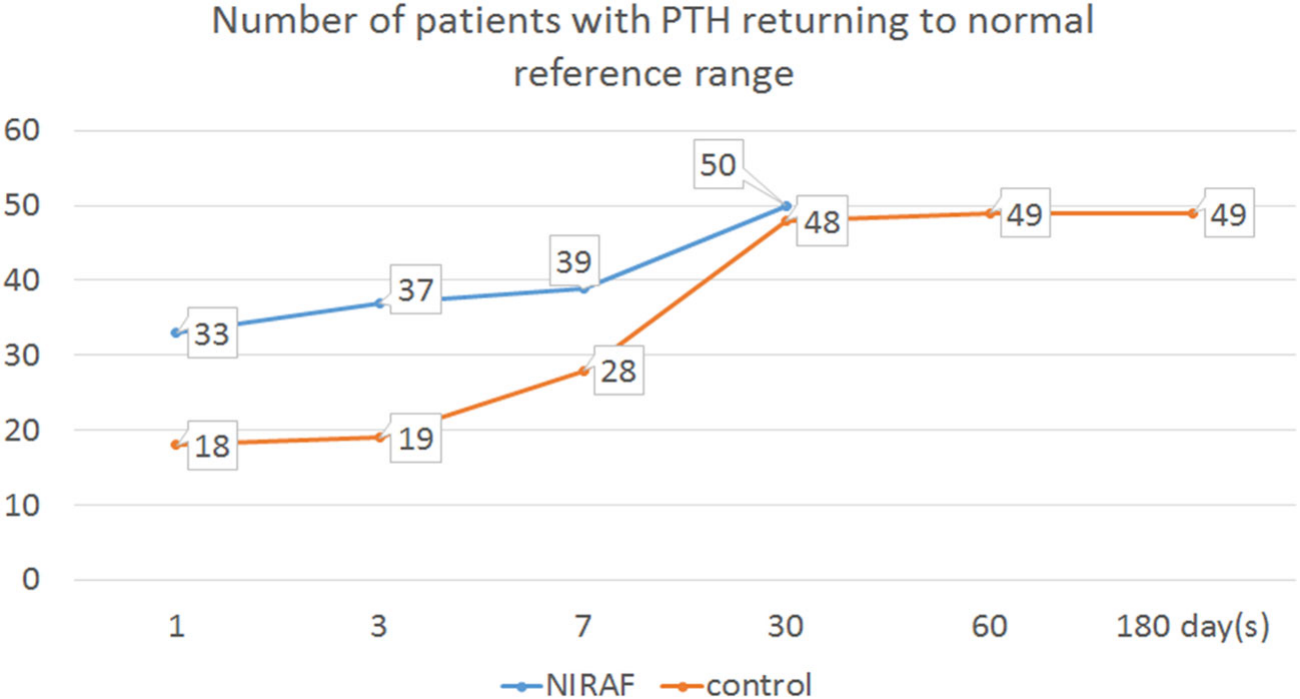
Time for PTH recovery to the normal reference range in the two groups after surgery.

On the third postoperative day, the PTH level in 74% of patients in the NIRAF group had recovered to normal levels, whereas only 38% of patients in the control group had recovered (p<0.001, χ^2 =^ 13.149). The PTH levels in all patients in the NIRAF group had recovered within 30 days after surgery, whereas those in the control group recovered more slowly. One patient in the control group failed to return to the normal level 6 months after surgery and required oral calcium supplementation to reduce the symptoms of fingertip numbness. That patient was diagnosed with permanent parathyroidism (**Figure 8**).

## 4 Discussion

Because the size, shape, color, and position of the parathyroid gland are variable, they are difficult to identify during surgery. In most cases, their identification relies on the surgeon’s experience. Various techniques have been used in the clinic to ascertain the development and localization of a parathyroid gland, including preoperative ultrasonic localization (15), radionuclide technetium 99m methoxyisobutyl isonitrile labeling, intraoperative Methylene Blue intravenous infusion or local staining (16, 17), 5-aminolevulinic acid fluorescence development (18),nanocarbon negative imaging (19), immunocolloidal gold technology (20), and other detection techniques. The most frequently used intraoperative identification techniques need to input exogenous substances into the human body or are contact-invasive operations, which are time-limited and vulnerable to the influence of local blood supply and scarring, thus it is difficult to meet the requirements of real-time and repeatable imaging.

After Paras et al. (21) reported that fluorescent signals could be detected from parathyroid glands using near-infrared light, this non-invasive method has been continuously improved. The principle is to use an approximately 15 kDa fluorophore with an unknown structure in parathyroid cells to generate 820 nm autofluorescence (22) under 785 nm wavelength light. This technique does not require the injection of other positive/negative developers; it reduces the possibility of allergic reactions and the interference of local drugs on the operation field, and is not affected by a wound scar or blocked lymphatic drainage. Secondly, NIRAF imaging can be performed at any time and repeatedly; therefore, there is little interference with the surgical procedure. Finally, NIRAF identifies specific fluorescent substances in both *in situ* and *in vitro* parathyroid glands. It can assist doctors in saving inadvertently resected parathyroid glands before the specimens are sent for pathological examination.

Randomized controlled trials explored the role of NIRAF in total thyroidectomy and demonstrated that it can effectively improve the detection rate and reduce the incidence of hypocalcemia (23, 24). However, not all cases included in those studies required bilateral lymph node dissection; thus, it is only necessary to ensure that the parathyroid glands around the thyroid gland are not accidentally cut off during surgery. Clinically, patients who underwent total thyroidectomy and bilateral central neck dissection have a greater risk of postoperative hypoparathyroidism. To ensure that parathyroid glands are not resected, while lymph nodes are completely removed, doctors are required to identify each parathyroid gland accurately and ensure that its function is intact after surgery. Our step-by-step method provides a strategy for locating and protecting all non-ectopic parathyroid glands.

Strategies for *in situ* retention of parathyroid glands have protected the gland structure and entered the next stage: the protection of gland function. Compared with the thyrothymic ligament type C3 and D3 glands and the intrathymic type C5 and D5 glands, in the first instance, doctors may prefer to encounter compact parathyroid glands (types C1, D1, C2, and D2) as they are easier to identify when dissociating the thyroid gland. With the popularization of meticulous capsule dissection techniques, most doctors can identify and retain more than two intact parathyroid glands during surgery, and the incidence of postoperative permanent parathyroidism has decreased significantly. At this point, reduction in temporary parathyroidism has become the goal of more refined surgery. In addition to structural preservation, the protection of parathyroid function (blood supply) is also very important. The blood supply to compact parathyroid gland is closely associated with that of the thyroid gland and is easily damaged. In some cases, the bilateral superior and inferior parathyroid glands are retained in situ; however, serious parathyroidism may still occur after surgery.

In addition to structural protection, protection of parathyroid function was the main consideration, and doctors used techniques such as intraoperative indocyanine-green (ICG) to evaluate the blood supply to the parathyroid glands and guide autologous transplantation (25). However, the most effective way to avoid postoperative parathyroidism is to identify and protect the vascular network around the parathyroid glands during surgery (26). Because the position of the parathyroid gland (especially the inferior parathyroid gland) is not fixed, even experienced doctors often face the problem that, after accurately identifying the parathyroid gland, they find that its blood supply has been damaged during the earlier dissection and can only choose autologous transplantation. Although some classifications of parathyroid glands have been reported (14, 27), guidance for clinical operations is limited. Our classification and surgical procedure can first locate the parathyroid gland before dissection and then retrograde dissect its blood supply vessels to the trunk to better protect the blood supply to the parathyroid gland. It can show the possible location of the parathyroid gland in a certain area on the premise of less disturbance of the local vascular network and then the next possible area can be entered with an undamaged vascular network.

In the NIRAF group, we found that more than 95% of the superior parathyroid glands and more than 85% of the inferior parathyroid glands were identified before the dangerous phase, which was much higher than that in the control group. No serious long-term parathyroidism was observed in the NIRAF group. The incidence of postoperative temporary parathyroidism and hypocalcemia in the NIRAF group was also lower than that in the control group, and postoperative PTH recovery in the NIRAF group was faster than that in the control group. In the control group, we found that, although the compact parathyroid glands were easy to identify, the blood supply might originate from the branches on the surface of the thyroid gland and these were susceptible to inadvertent disconnection when the capsule was dissected. In the NIRAF group, the parathyroid gland was located first, and its blood vessels could be dissected along the proximal direction, which could greatly improve the protection of the blood supply. For other non-compact parathyroid glands, although their blood supply was distant from the thyroid gland and easy to retain, it was difficult to locate and expose them with only the naked eye. They were sometimes found during specimen examination, or even during postoperative paraffin pathology. In the NIRAF group, we found that the location of most non-compact parathyroid glands could be identified before thyroidectomy or neck dissection was completed. Therefore, this method has obvious advantages in terms of protecting the structure and function of these glands.

This study also has some limitations. The number of cases is small and it is a study from a single center and the same team. Secondly, the cases included in the study were all early tumors. For the cases with locally advanced tumors invading the capsule extensively, the parathyroid gland may be involved and difficult to image. In fact, it is more difficult to protect the parathyroid gland in the circumstances. Some of them can refer to our above procedures, while the tumor involved parts may require individualized treatment. Finally, we did not perform ICG imaging. Therefore, it is difficult to directly evaluate the blood supply of parathyroid glands, which can only be indirectly reflected by the postoperative PTH level. In the future, we will further expand the sample size and supplement relevant experiments.

## 5 Conclusion

The step-by-step near-infrared autofluorescence parathyroid identification method can be used to effectively locate parathyroid glands and protect their function. This method may improve the *in situ* preservation rate of parathyroid glands and further reduce the occurrence of temporary parathyroidism after surgery. To reduce inadvertent interference of the parathyroid glands during surgery and improve their detection rate, we summarize the steps as follows: P1, lateral dissection of the thyroid; P2, dissection of the thyrothymic ligament; P3, ligation of tertiary vessels on the thyroid surface; P4, removal of the thyroid gland; P5, central neck dissection; and P6, examination of collected specimens.

## Data availability statement

The original contributions presented in the study are included in the article/supplementary materials, further inquiries can be directed to the corresponding author/s.

## Ethics statement

The studies involving human participants were reviewed and approved by The Ethics Committee of Beijing Tongren Hospital, Capital Medical University. The patients/participants provided their written informed consent to participate in this study.

## Author contributions

Drafting the manuscript: JH, QZ. Acquisition of data: XC, YZ, XHC, JF. Analysis of data: JH, QZ, YZ. Revision of the manuscript for important intellectual content: YH, YW, YZ. Conception and design of the study: JH, QZ. All authors contributed to the article and approved the submitted version.

## References

[1] Kim J Gosnell JE Roman SA. Geographic influences in the global rise of thyroid cancer. Nat Rev Endocrinol (2020) 16(1):17–29. doi: 10.1038/s41574-019-0263-x 31616074

[2] Du L Li R Ge M Wang Y Li H Chen W. Incidence and mortality of thyroid cancer in China, 2008-2012. Chin J Cancer Res (2019) 31(1):144–51. doi: 10.21147/j.issn.1000-9604.2019.01.09 PMC6433579 30996572

[3] Wang J Yu F Shang Y Ping Z Liu L. Thyroid cancer: incidence and mortality trends in China, 2005-2015. Endocrine (2020) 68(1):163–73. doi: 10.1007/s12020-020-02207-6 32002755

[4] Puxeddu E Tallini G Vanni R. What is new in thyroid cancer: The special issue of the journal cancers. Cancers (Basel) (2020) 12(10):E3036. doi: 10.3390/cancers12103036 PMC7603182 33086491

[5] Papaleontiou M Hughes DT Guo C Banerjee M Haymart MR. Population-based assessment of complications following surgery for thyroid cancer. J Clin Endocrinol Metab (2017) 102(7):2543–51. doi: 10.1210/jc.2017-00255 PMC5505192 28460061

[6] Teshima M Otsuki N Morita N Furukawa T Shinomiya H Shinomiya H. Postoperative hypoparathyroidism after total thyroidectomy for thyroid cancer. Auris Nasus Larynx (2018) 45(6):1233–8. doi: 10.1016/j.anl.2018.04.008 29747960

[7] Vasileiadis I Karatzas T Charitoudis G Karakostas E Tseleni-Balafouta S Kouraklis G. Association of intraoperative neuromonitoring with reduced recurrent laryngeal nerve injury in patients undergoing total thyroidectomy. JAMA Otolaryngol Head Neck Surg (2016) 142(10):994–1001. doi: 10.1001/jamaoto.2016.1954 27490310

[8] Orloff LA Wiseman SM Bernet VJ Fahey TJ 3rd Shaha AR Shindo ML. American Thyroid association statement on postoperative hypoparathyroidism: Diagnosis, prevention, and management in adults. Thyroid (2018) 28(7):830–41. doi: 10.1089/thy.2017.0309 29848235

[9] Voelker R. Devices help surgeons see parathyroid tissue. JAMA (2018) 320(21):2193. doi: 10.1001/jama.2018.18768 30512085

[10] Kose E Rudin AV Kahramangil B Moore E Aydin H Donmez M. Autofluorescence imaging of parathyroid glands: An assessment of potential indications. Surgery (2020) 167(1):173–9. doi: 10.1016/j.surg.2019.04.072 31526579

[11] Demarchi MS Seeliger B Lifante JC Alesina PF Triponez F. Fluorescence image-guided surgery for thyroid cancer: Utility for preventing hypoparathyroidism. Cancers (Basel) (2021) 13(15):3792. doi: 10.3390/cancers13153792 34359693 PMC8345196

[12] Kim DH Lee S Jung J Kim S Kim SW Hwang SH. Near-infrared autofluorescence-based parathyroid glands identification in the thyroidectomy or parathyroidectomy: a systematic review and meta-analysis. Langenbecks Arch Surg (2022) 407(2):491–9. doi: 10.1007/s00423-021-02269-8 34322746

[13] Solórzano CC Thomas G Baregamian N Mahadevan-Jansen A. Detecting the near infrared autofluorescence of the human parathyroid: Hype or opportunity? Ann Surg (2020) 272(6):973–85. doi: 10.1097/SLA.0000000000003700 PMC8670620 31804401

[14] Perrier ND Edeiken B Nunez R Gayed I Jimenez C Busaidy N. A novel nomenclature to classify parathyroid adenomas. World J Surg (2009) 33(3):412–6. doi: 10.1007/s00268-008-9894-0 19148701

[15] Shou JD He SM Jiang XF Shi LH Xie L Wang JB. Anatomical localization of normal parathyroid glands before thyroidectomy through ultrasonography reduces postoperative hypoparathyroidism. Med (Baltimore) (2019) 98(24):e16020. doi: 10.1097/MD.0000000000016020 PMC6587641 31192951

[16] Patel HP Chadwick DR Harrison BJ Balasubramanian SP. Systematic review of intravenous methylene blue in parathyroid surgery. Br J Surg (2012) 99(10):1345–51. doi: 10.1002/bjs.8814 22961511

[17] Piromchai P Juengtrakool T Laohasiriwong S Kasemsiri P Ungarereevittaya P. The sensitivity and specificity of methylene blue spray to identify the parathyroid gland during thyroidectomy. PeerJ (2019) 7:e6376. doi: 10.7717/peerj.6376 30697498 PMC6347963

[18] Elbassiouny S Fadel M Elwakil T Elbasiouny MS. Photodynamic diagnosis of parathyroid glands with nano-stealth aminolevulinic acid liposomes. Photodiagnosis Photodyn Ther (2018) 21:71–8. doi: 10.1016/j.pdpdt.2017.11.004 29155074

[19] Li Y Jian WH Guo ZM Li QL Lin SJ Huang HY. A meta-analysis of carbon nanoparticles for identifying lymph nodes and protecting parathyroid glands during surgery. Otolaryngol Head Neck Surg (2015) 152(6):1007–16. doi: 10.1177/0194599815580765 25897006

[20] Zhang A Gao T Wu S You Z Zhen J Wan F. Feasibility of using colloidal gold immunochromatography for point-of-care identification of parathyroid glands during thyroidectomy. Biochem Biophys Res Commun (2018) 507(1-4):110–3. doi: 10.1016/j.bbrc.2018.10.178 30420286

[21] Paras C Keller M White L Phay J Mahadevan-Jansen A. Near-infrared autofluorescence for the detection of parathyroid glands. J BioMed Opt (2011) 16(6):067012. doi: 10.1117/1.3583571 21721833

[22] Ladurner R Al Arabi N Guendogar U Hallfeldt K Stepp H Gallwas J. Near-infrared autofluorescence imaging to detect parathyroid glands in thyroid surgery. Ann R Coll Surg Engl (2018) 100(1):33–6. doi: 10.1308/rcsann.2017.0102 PMC5838663 29022781

[23] Dip F Falco J Verna S Prunello M Loccisano M Quadri P. Randomized controlled trial comparing white light with near-infrared autofluorescence for parathyroid gland identification during total thyroidectomy. J Am Coll Surg (2019) 228(5):744–51. doi: 10.1016/j.jamcollsurg.2018.12.044 30710614

[24] Benmiloud F Godiris-Petit G Gras R Gillot JC Turrin N Penaranda G. Association of autofluorescence-based detection of the parathyroid glands during total thyroidectomy with postoperative hypocalcemia risk: Results of the PARAFLUO multicenter randomized clinical trial. JAMA Surg (2020) 155(2):106–12. doi: 10.1001/jamasurg.2019.4613 PMC6865247 31693081

[25] Jin H Cui M. Research on intra-operative indocyanine green angiography of the parathyroid for predicting postoperative hypoparathyroidism: A noninferior randomized controlled trial. Endocr Pract (2020) 26(12):1469–76. doi: 10.4158/EP-2020-0340 33471739

[26] Hou D Xu H Yuan B Liu J Lu Y Liu M. Effects of active localization and vascular preservation of inferior parathyroid glands in central neck dissection for papillary thyroid carcinoma. World J Surg Oncol (2020) 18(1):95. doi: 10.1186/s12957-020-01867-y 32404116 PMC7222446

[27] Zhu J Tian W Xu Z Jiang K Sun H Wang P. Expert consensus statement on parathyroid protection in thyroidectomy. Ann Transl Med (2015) 3(16):230. doi: 10.3978/j.issn.2305-5839.2015.08.20 26539447 PMC4598451

